# Exploring the acceptability of remote care for people with psychotic disorders in the community: practical challenges and desired features

**DOI:** 10.3389/fpsyt.2025.1409455

**Published:** 2025-11-03

**Authors:** Nadia Abdel-Halim, Ronja Kuhn, Sarah Bourdin, Patrick Healey, Victoria Bird, Philip McNamee

**Affiliations:** ^1^ Unit for Social and Community Psychiatry, East London NHS Foundation Trust, London, United Kingdom; ^2^ Unit for Social and Community Psychiatry, Wolfson Institute of Population Health, Queen Mary University of London, London, United Kingdom; ^3^ School of Electronic Engineering and Computer Science, Queen Mary University of London, London, United Kingdom; ^4^ Institute of Public Health & Wellbeing, University of Essex, Essex, United Kingdom

**Keywords:** remote care, therapeutic relationship, accessible care, psychosis, user design

## Abstract

**Introduction:**

People with psychosis are more likely to experience paranoia, which can be worsened by technology use, and have lower digital literacy comparative to the general population and other serious mental illness populations (e.g., recurrent depression). The expedited uptake of remotely delivered mental healthcare by secondary mental health services in the UK in recent years (most notably during the height of the COVID-19 pandemic) provides an opportunity to understand how service users with psychosis, and their clinicians, view the impact of rising digitization within mental health services. This study aimed to explore the present factors encouraging and discouraging the use of remote care options through using the example of a face-to-face intervention, DIALOG+, currently being developed to be delivered remotely. A secondary objective was to identify which requirements need to be met for clinicians and service users to willingly adopt novel remote interventions.

**Methods:**

9 workshop-focus groups were conducted in total, 6 with service users with psychosis (n= 17) and 3 with clinicians (n=21). Participants were recruited from community mental health services from urban (East London) and rural (Cornwall) settings so that comparisons could be made across contexts. Workshop-focus groups were recorded and transcribed before analysis using the framework method.

**Results:**

The analysis identified four overarching themes: Factors influencing the acceptability of remote care; Adaptability of remote care for inclusivity; Influence of remote care on therapeutic relationships; and Desirable features in remote care.

**Discussions:**

Service users and clinicians did not view remote delivery of care as a completely adequate substitute for in-person care but were receptive to its integration into hybrid models when both patient choice and clinician judgment were respected. The study highlights the need for software design to address resource limitations and individual differences, while considering the influence of training, device availability, and urbanization on remote care implementation.

## 1 Introduction

The integration of digital and remote delivery of mental healthcare within the UK National Health Service (NHS) has been a national and strategic focus since the publication of the NHS Long Term Plan in 2019, however, the COVID-19 pandemic accelerated the use of digital technology in healthcare at a time when social distancing was mandatory (1, 2). Digital transformation is a key component of the 10-year NHS Long Term Plan, and despite the slow uptake of telehealth and remote care options within secondary mental health, it is a priority for the NHS to make better use of technology and innovation to streamline and improve service delivery in the UK (3). Despite these laudable aims, the rise of digitalization and remote mental health care delivery has not always been readily welcomed by either clinicians and service users with severe mental illnesses, particularly those with psychosis for myriad reasons (4). For example, people with psychosis face particular challenges relating to remote care such as persecutory delusions and/or hallucinations in relation to technology use, and a greater apprehension toward technology than the overall population (5, 6). It is therefore essential to co-produce approaches to remote delivery with service users and clinicians so as not to further marginalize populations with psychosis and increase health inequities.

There have been numerous cited benefits to remote care adoption within mental health care and healthcare more generally. The cost-effectiveness of remote mental healthcare options has been established across various modalities including video monitoring, text messaging interventions, and digital health portals (7, 8). Evidence of reduced appointment wait times and overall cost reductions within primary care have also been generalized to mental healthcare contexts (9). Service users accessing remote care have been found to have more flexibility and diminished journey costs and travel times to mental health clinics, enabling them to better manage their treatment within the limitations that childcare or work responsibilities might pose (10, 11). More recently, there has also been a push within mental healthcare to use digital health portals to improve transparency of care, providing service users with a novel tool to view their own patient records with fewer bureaucratic obstacles (12).

However, clinicians have cited several issues with remote care delivery such as diminished control and insight during therapy sessions, difficulties with disconnection and safeguarding concerns (13). Similarly, service users have noted that they can struggle to build rapport with clinicians online and often feel distressed due to paranoia, that can be worsened by technology use (14). While, governmental social distancing policies in reaction to the COVID-19 pandemic led to the acceleration of remote care adoption by the NHS, policy directives for how to use and anticipate barriers to remote delivery were often developed retroactively and clinicians received limited training on remote best-practice guidelines (15, 16).

The previous literature on remote mental healthcare for people with psychosis has largely been experimental in nature, studying the efficacy of specific digital applications or tools on preselected outcomes. While many studies boast positive experiences of remote care, short follow-up periods may explain the gaps between the positive care experiences reported by studies and the concerns reported by service users and clinicians using remote care options in practice (10, 17). The COVID-19 pandemic, where many service users accessed remote mental health care, has provided an important opportunity to better understand the realities of remote care delivery within secondary mental health services and how these experiences have impacted the willingness or apprehension of clinicians and service users to engage in remote mental healthcare. Further, it has highlighted the difficulties and barriers of developing and delivering remote care and how healthcare providers can avoid these pitfalls in the future. This study therefore aims to identify the present factors encouraging and discouraging the use of remote mental healthcare options for those with psychosis, as well as which requirements need to be met for clinicians and service users for them to willingly adopt novel remote mental health interventions.

## 2 Materials and methods

### 2.1 Design and setting

The present study had an exploratory qualitative design, using “workshop-focus groups” to elicit perspectives on remote mental health delivery from service users with psychosis and the clinicians who treat them in community-based settings. Workshops as a research methodology have been increasingly used in recent years whereby a domain-related research question uses a “workshop” format to fulfil a research purpose and to produce reliable and valid data about the domain in question (18). This combined with established focus group methods to produce interactive data from multiple participants, aimed to generate insightful and meaningful data.

The study was conducted at two NHS Trusts: Cornwall Partnerships NHS Foundation Trust and East London NHS Foundation Trust, both located in the South of England. These study sites were specifically selected to improve understanding of the differing needs and considerations required for urban (East London) and rural (Cornwall) contexts.

The study received favorable ethical opinion from the North West- Preston Research Ethics Committee (Ref: 22/NW/0018) and was approved by the Health Research Authority (HRA).

The research aims and materials were reviewed by a lived experience panel of people with psychotic disorders and their carers. The protocol and materials were adapted following their feedback.

### 2.2 Recruitment and eligibility criteria

Purposive sampling was used to recruit service users and clinicians within NHS community mental health services.

Clinicians were recruited directly by researchers using established networks. Service users with psychosis were identified by their treating clinician, and then once researchers received permission to contact them, a member of the research team provided additional study information and collected informed consent, either over the phone or face to face.

Service users were eligible for participation if they were over the age of 18, had the mental capacity to consent, had spoken command of English, and had received a diagnosis of psychosis, defined as any F20–29 diagnosis within the International Classification of Diseases-10 (ICD-10)- this includes schizophrenia, schizotypal, delusional, and other non-mood psychotic disorders. As psychotic disorders have high comorbidity with other mental health conditions, service users with multiple psychiatric conditions were not excluded and were deemed eligible to participate.

Clinicians were eligible if they were clinical staff, aged 18 or older, and currently working within a community mental health service that had contact with service users who have a diagnosis of a psychotic disorder or had six months previous experience working in such teams. Non-clinical staff were excluded.

### 2.3 Data collection

Data was collected between April 2022 and December 2022. Prior to each workshop-focus group, individual informed consent was obtained, and demographic information was collected from each participant by the study research assistants (NA-H & KE). Upon approach, potential participants were informed of the objectives of the research through a Participant Information Sheet.

Sessions were conducted online using Microsoft Teams or in-person to accommodate different participant preferences. All workshop-focus group sessions were audio-recorded (if held in person) or screen-recorded (if held online). Allowing participants to have a choice between these two settings was done to encourage the engagement of participants with varying levels of digital literacy and confidence in using communication software. Workshop-focus groups were scheduled flexibly, including outside regular working hours to reduce barriers to involvement for those with scheduling commitments during the standard working hours of 9am to 5pm.

The workshop-focus groups lasted between one hour and two hours depending on the number of participants involved in each group with a pre-determined maximum of 8 participants per focus group. Service user participants were called before the focus group to discuss and address any accessibility needs. All participants completing the workshop-focus groups remotely were encouraged to join the session from an area that they would be most likely to join a remote clinical care session from. This was done to facilitate reflections on what challenges the environment might present to online care. Focus groups were one off, and no repeat data collection took place.

Workshop-focus group sessions for both clinicians and service users began with a 10-minute didactic workshop about remote care followed by a focus group discussion. The workshop briefly defined remote care, based on current definitions, and introduced the DIALOG+ intervention. DIALOG+ is a technology-assisted and resource- oriented intervention which attempts to structure communication between service users and their clinicians during routine meetings in mental health care settings, aiming to create better treatment plans and improve clinical outcomes (19). It was originally developed for people with psychosis. This real-world example was presented to help structure thinking around how an existing therapeutic intervention may be modified or adapted to remote delivery, and what some of the key considerations may be. The participants were also asked to share what their own understandings of remote care may be. This was then followed by a focus group discussion lasting 50–90 minutes. Only participants and researchers were present at the focus groups, there were no observers or non-participants. Research assistants used clinician and service user participant topic guides to probe the perspectives of participants on the following key topics: personal access to digital devices and internet connection for remote care; necessary training, and preparation for delivery/receipt of remote care; integration of remote care within daily routine; privacy and confidentiality considerations; past experiences of remote care; and overall impact of remote care on the therapeutic alliance. While both guides both explored the same areas (see **Supplementary Material Figure 1**), the clinician topic guide (see **Supplementary Material Figure 2**) was adapted to include more questions regarding the practical challenges of online communication to therapeutic effectiveness. Both the presentation and the topic guide were reviewed by an involvement panel of service users with lived experience of psychosis prior to data collection, to ensure that it would be fit for purpose and that the language used was appropriate, sensitive, and comprehensible.

### 2.4 Data analysis

The focus group recordings were transcribed verbatim, ensuring participant details were pseudonymized at this stage, replacing all identifying information, including names and locations of mental health care teams. Transcripts were not returned to participants for comments. Once transcribed, transcripts were cleaned and underwent a thematic analysis following the framework method described by Gale and colleagues (20). Themes were derived from the data, and not pre-determined. This analysis was undertaken independently by two female members of the research team, who were employed as research assistants on the study, and had Masters level education in research methods (NA-H, RK). The researchers first read three transcripts and familiarized themselves with their content. Then, each researcher free-coded the transcripts before meeting to discuss and compare the preliminary themes that they had identified. This discussion formed the basis for the development of a working analytical framework into which the data from every transcript was charted using Microsoft Excel.

After the framework matrix was completed with the coded and categorized data, analysis meetings were held with PM, a male academic psychologist with expertise (PhD) in qualitative research methods to interpret the data and map connections and differences. Analytic memos or notes taken by the researchers throughout the analytic process were discussed and interrogated before the themes were finalized. Findings were discussed with wider members of the research team to provide alternative and multidisciplinary views when interpreting and developing the themes from the data.

Data saturation was not discussed within the team, and analysis stopped when the analysis team found the themes to be suitably inclusive and descriptive of the generated data. Participants did not provide feedback on the analysis, although preliminary versions of the matrix were presented to the Service User Design Panel.

## 3 Results

### 3.1 Sample demographics

Overall, the study recruited 17 service user participants (n=8 from Cornwall, n=9 from East London). There were 6 service user workshop-focus groups conducted in total, three from each study site. The focus groups in East London had 4, 2 and 3 attendees respectively and the Cornwall focus groups had 3, 3 and 2 attendees respectively. The average focus group size for service users was 2.8 participants.

The service users recruited to the workshop-focus groups were aged between 18 and 54, with most participants being between 35–44 years old (41%). 47% of service user participants were male and 35% of participants were female with the remaining two participants identifying as non-binary or preferring not to disclose. The majority of participants (88.2%) indicated that they were unemployed while the remaining 22.9% were in full-time employment. Notably, the research team made a concerted effort to offer timings outside of regular work hours for focus groups to involve participants who were in employment.

While the research team were able to recruit an ethnically diverse sample of participants, there were significant differences between the two NHS sites. These differences reflect the ethnic demographics of each area. Within Cornwall, 88.9% of participants identified as White British with the remaining participant identifying as Asian British. Meanwhile, half of the participants recruited from East London were White-British with the remaining four participants selecting their ethnicity as White-Other, British-Asian Bangladeshi, British-Asian Indian and Black British-African (respectively).

We recruited a total of 21 clinicians from the two participating mental health trusts (n=14 from East London and n=7 from Cornwall). Clinicians came from a diverse set of clinical roles; a list can be found in **Table 1** of the **Supplementary Material**.

There were 3 clinician workshop-focus groups conducted in total, two in East London (with each having n=7 attendees) and one in Cornwall (n =7). The two East London clinician workshop-focus groups were conducted online via MS Teams, and the Cornwall workshop-focus group was conducted face-to-face in a local village hall.

### 3.2 Themes

Based on the framework analysis, four themes were developed from the data. As data was thematically similar, themes were based on data from both service user and clinician participant groups and are presented together. Quotes are presented below with a pseudonym, gender and age range and if they were a service user or clinician and from which site they were recruited. The first theme, ‘Factors influencing the acceptability of remote care’ captured the general facilitators and barriers to the use of remote care. Theme 2 ‘Adaptability of Remote Care for Inclusivity’ investigated how remote care can be developed and amended to better meet the needs of individual service users and assist underrepresented groups to engage with their care. Theme 3, ‘Influence of Remote Care on Therapeutic Relationships’ goes beyond the suitability of remote care for the individual, to explore how remote care’s adoption impacts the service user’s ecological system, including their self-concept, their relationship with their therapist, and their relationship with the community. Finally, theme 4, ‘Desired Features’ uses a more applied lens to produce recommendations for remote care based on the features and functions that were requested by participants. **Table 2** in the **Supplementary Material** provides an overview and short description of all themes and subthemes.

#### 3.2.1 Theme 1: factors influencing the acceptability of remote care

##### 3.2.1.1 Willingness to engage in remote care

Service user and clinician participants both expressed that remote care is not a wholly adequate substitute for face-to-face care and as such, face-to-face care is necessary in some capacity for treatment delivery to be effective. However, participants noted that remote care does have a role in service delivery, and may be especially helpful for those facing obstacles to attending face-to-face treatment such as those with childcare responsibilities, social anxiety or social phobias. Participants also reflected on the utility of remote care in limiting time and transportation costs related to therapy.

While some participants noted that they may be initially resistant, most service users were largely open and willing to engage with remote care options, being particularly helpful and convenient when they felt more stable and had already built rapport with their clinician. Participants cited several instances where remote care would be preferable to in-person care including being abroad or otherwise being unable to come into clinics, or in circumstances of isolation due to public health measures or ill health when service users are unable to join in-person. Participants with neurodivergent conditions such as autism also explained that remote care options allowed them to better manage sensory difficulties which can be distracting during in-clinic care.

“I feel like it’s better as a backup. I wouldn’t want my whole treatment plan to become online- an app, but I think it’s definitely a useful thing to have as a backup or as an addition.” *– Regina, 18-24, Woman, Service User, Cornwall*

Service user participants highlighted that it would not be suitable for diagnostic processes due to symptoms being more easily masked online and clinician intuition being compromised. Those who were opposed to adopting remote care cited previous negative experiences including misdiagnosis or reluctance to change existing care arrangements.

Clinicians were largely willing to use remote care but identified a need for improved guidance on how to do so in accordance with best practice recommendations. Clinicians also highlighted that they would need their ability to exercise clinical judgment to not be hampered by pressure to provide online services, noting that the push toward online care needs to be driven by accommodating service user demands, not economic arguments.

##### 3.2.1.2 Access to devices and connectivity

All participants who attended a focus group had access to a smartphone or were confident that they could ask someone they lived with to borrow one. However, participants were hesitant to access their care in this way, citing paranoia related to the device use or discomfort holding the device or viewing their clinician on a smaller screen. Internet connectivity was another major concern for participants, particularly in rural settings.

Participants from Cornwall noted the existence of digital not-spots such as “the dip” in Penrith where the absence of cellular or internet connection would make it impossible for some residents to connect from their phones. Furthermore, participants in East London noted that group living facilities are often not equipped with Wi-Fi internet, making it impossible to receive remote care without facing financial boundaries.

“When I was living in this temporary accommodation which was when I needed service the most, there was actually no internet in the facility. So I was on a £20 per month data package to try and get me enough internet to exist really.*” – Louis, 25-34, Non-Binary, Service User, East London*

Difficulties with accessing the internet for sessions was not limited to service users. Service users in Cornwall noted that even when their internet connection was relatively stable, that of their clinicians was not. Clinicians in both East London and Cornwall noted that they found the internet within NHS buildings unreliable and were often fearful about being disconnected in the middle of sessions.

##### 3.2.1.3 Digital literacy and access to support

Service users spoke about the lack of support for those not confident in using digital technologies. They noted that the increased digitalization of key processes such as scheduling appointments, neglects service users who may not understand how to access their emails and do not have the digital skills to download and use specific applications.

Service users and clinicians both emphasized the need for improved digital support for remote care to be considered a viable option. Some service users felt that they would benefit from formal training in how to use specific digital health interventions, noting that this could be facilitated in a group format to foster a supportive space where service users could meet and help one another. Service users noted that it was important for any training to acknowledge symptomatic issues such as paranoia and confidentiality concerns. Despite an interest in building digital competencies, service users also noted that being provided with too much training might deter them from using the intervention.

“Maybe people will get ‘the off’ by that … because they have an app and then you’re telling them to go watch a video of how to use it and maybe they don’t have the time to do that.” *– Luther, 25-34, Man, Service User, East London*

Clinicians expressed a similar frustration surrounding training, noting that they are often bombarded with mandatory training or training manuals that are difficult to navigate and offer inadequate practical advice on how to resolve common issues with the technology.

“It needs to be live and if we do need a dummy’s guide, it’s better to embed that in the software so if you don’t know what you’re doing, go to the three buttons at the top, go to menu and just click and then there should be one page and that’s it. But please don’t give us any paper, you’re just going to kill trees for no reason.” *– Andrea, Woman, Operational Lead, East London*

##### 3.2.1.4 Privacy, security and safety

Service users were largely trusting of NHS applications, explaining that they were aware of the strict information governance requirements for their information to be shared with any third parties. Meanwhile, clinicians emphasized the importance of balancing the need for confidentiality precautions with the ability to work flexibly. Clinicians explained that employing secure remote access software that was introduced by NHS Trusts during the COVID-19 pandemic could be time-consuming and often hampered their ability to join sessions rapidly or respond dynamically to service user requests.

Despite service user trust in existing security measures, many voiced that they did not have a private space from which they could join their sessions and ensure their own confidentiality. Participants living with family or in shared accommodation stated that this would likely affect what they disclosed or discussed with their clinician,

“I think it would affect, for example (…) I’m talking about relationships and my boyfriend is also hearing it might affect how much I would be able to share with my therapist clinician. And if I’m talking about my parents and my parents are also in my house it would affect the amount of information I would be sharing.” *– Joy, 18-24, Woman, Service User, East London*

Moreover, service users and clinicians raised the difficulties that remote care posed to the completion of accurate risk assessments. Both groups commented on the potential for abusive individuals in the home to render remote-therapy sessions an unsafe space by compromising the service user’s assurance of privacy and security.

Clinicians noted safeguarding concerns pertaining to what may or may not be visible in the service user’s environment. This led to a sense of hypervigilance for the clinicians which became exhausting, leading to a cognitive load which discouraged them from engaging in remote care options. Beyond this, clinicians were also concerned about their own privacy and security of information, explaining that often video-conferencing platforms provide service users with access to their emails or phone numbers which removed a professional boundary and went against service policies.

Clinicians also shared experiences of having had their sessions recorded without their consent by service users online,

“Learning from our current situation, people tend to record us without telling us they’re recording us. And sometimes they bring in an entire group of people to listen in and that’s fine if you’ve got nothing to hide … but as a practitioner, you have to be quite mindful because I’ve dealt with a number of complaints … Are you [the patient] happy for them to be there? Because we may be dealing with any kind of domestic violence issues, we always have to be mindful of that safeguarding concern.” – *Andrea, Woman, Operational Lead (Clinician), East London*

Therefore, the need for privacy, confidentiality and safety is a salient concern for both service users and clinicians.

##### 3.2.1.5 Previous experiences

Service users’ willingness to use remote care was largely informed by their previous experiences of using other remote care software, particularly during the COVID-19 pandemic. Service users who had negative experiences of remote care stated that this impacted their willingness to engage in other remote care options.

“I think with such big diagnosis. I would rather have seen someone than do it over some technology. It just didn’t feel right. I didn’t get the care that I should have got, in my opinion … he didn’t see my symptoms cause obviously it was technology. So, in a face-to-face consultation, they would have picked it up and gave me the right medication, not made myself worse.” *– Nooriyah, 35-46, Woman, Service User, East London*

Conversely, other service users explained that the shift to remote care during the pandemic was beneficial in some ways, particularly in regard to removing the need to travel and allowing one to be in their own environment,

“I feel more comfortable, it helps me to open up as I am in my own environment in my own surroundings rather than having to travel, going to a place, being late which adds even more stress and anxiety to how I am already feeling.” *– Radwan, 35-44, Man, Service User, East London*

Similarly, clinicians in both localities found that the forced trial of remote care facilitated by the pandemic allowed them to challenge their own, initially negative, conceptions of remote care and its viability.

So that was kind of a nice tool actually. And that’s what I didn’t think would work (…) during the pandemic I had to do it certain times and actually I was quite surprised how well we work together online. I just never thought you could do therapy online.” *– Samia, Woman, Team Manager (Clinician), Cornwall*.

#### 3.2.2 Theme 2: adaptability of remote care for inclusivity

##### 3.2.2.1 Appropriateness of remote care across illness severity

Service users remarked that the introduction of remote care is not always appropriate for those with psychosis who are experiencing delusions or auditory hallucinations which may be exacerbated by the use of technology. One service user explained how accessing remote care in the past had worsened his paranoia leading to him spending lots of time researching the privacy policies of common video-conferencing platforms. Another service user commented on the complicated relationship between psychosis and technology,

“When I came out of hospital, I really didn’t touch a phone and I didn’t wanna look at TV because I didn’t trust my own judgment. And so technology is a big thing in psychosis … Technology is really psychosis enemy*” – Clément, 25-34, Man, Service User, East London*

Service users explained that their auditory hallucinations and persecutory delusions often led them to feel that they were unsafe during their sessions, and that avoidance of technology following relapse followed by its slow re-integration was often incorporated into their treatment plans. Clinicians were aware of the complicated relationship that this clinical population has with technology and described how they often offered phone contact without video to accommodate this when clinically appropriate.

“It does depend on the person. With service users that I’ve been working with a long time, when I know them quite well, I find that phone contact is sufficient but there are some service users who wouldn’t want to do camera, if it’s to do with their diagnosis, maybe paranoia, a lot of stuff can be wrapped up in electronics and even using the phone can trigger a bit of paranoia” *-Maria, Woman, Mental Health Social Worker (Clinician), East London*

Therefore, the potential of remote care to exacerbate existing positive symptoms needed to be considered thoroughly and that patient choice in this matter was paramount.

##### 3.2.2.2 Adjustments to remote care for people with multiple conditions

Throughout the focus group interviews, service users noted that remote care could provide an avenue to care to those who cannot access conventional face-to-face treatment. Service users noted that those with physical disabilities or mobility issues might find it easier to join online than to travel.

Two service user participants from East London had a secondary diagnosis of autism and shared that remote care allowed them to have greater control over the sensory elements of the interaction, such as the volume of their therapist’s speech, providing them with a sense of control which enhanced their ability to engage.

Overall, service users were adamant that any remote care tools should consider the various comorbidities that people may have which could hamper their ability to use digital mental health interventions and noted that disabled users needed to be engaged proactively as opposed to workarounds being developed retroactively.

Similarly, a service user from Cornwall noted that remote care apps should cater to different people’s cognitive styles and learning preferences to improve their overall understanding of their care,

“I think simple steps maybe with visuals next to it that works for both people who are very good at kind of the auditory and some people work with visual. And so like maybe like words or sound or depending on the person” *– Stuart, 18-24, Non-Binary, Service User, Cornwall*

##### 3.2.2.3 Importance of simplified language and user-friendly design

Across both study sites, service users emphasized that the language used on any mobile application should be simplified to allow service users to understand the session agenda and better engage in their sessions. Service users emphasized that seeing jargon on their screen would potentially distress them and discourage them from using remote care, especially in periods of crisis.

“I think the apps need to be simplified anyway they shouldn’t be very hard and full of terminology and all that. I don’t think that’s nice when you’re ill and have that on you, it is another pressure.” *– Nooriyah, 35-46, Woman, Service User, East London*

Similarly, clinicians noted the potential of well-constructed and user-friendly designs in improving the efficiency with which care can be delivered. One clinician noted,

*“*I have been using Accurx for the majority of my sessions recently and it seems to be working really well … even people who seemed quite digitally naive have been able to do it cause it’s really easy to use. And so far I’ve not had any complaints and it’s meant that I can speed up delivering sessions and not having to wait around for rooms and things like that” *– Helga, Woman, Clinical Psychologist (Clinician), East London*

When asked about who remote care was most suitable for, clinicians identified that while differences in digital literacy exist across cultural and age groups, if the application is easy to follow, it is possible to engage a wider range of service users.

##### 3.2.2.4 Cultural inclusivity and the importance of including non-English speakers

Service user participants noted that cultural inclusivity was imperative to their decision to engage with remote care. These discussions largely dominated the focus groups in East London where service users shared their considerable concern about already pervasive difficulties in accessing culturally sensitive clinicians and requesting access to appropriate interpreters during in-person care. Service users expected such issues to be exacerbated if sessions moved online.

Service users felt that cultural competency needed to be used, to avoid misinterpretation, or worse, pathologization of some behaviors, when working remotely.

“It’s going to affect different languages and different cultures. Like with me [who’s] got an African background. My mom and my dad- they speak kinda louder. So if you don’t know that, like if the doctor was speaking to them, they’ll think it might be aggressive and stuff like that. But it’s just the way we speak” *– Clément, 25-34, Man, Service User, East London*

Service users who are not using an interpreter but have limited English noted their feeling of discouragement from therapy when being misunderstood during face-to-face sessions, especially when disclosing information which is emotionally salient or difficult to repeat. They suggested their ability to use body language to communicate would be hampered during a remote care session. Service users expressed that remote software should be translated into as many languages as possible to mitigate potential communication concerns.

Clinicians were similarly concerned about their ability to effectively communicate with limited English online. One clinician stated,

“Sometimes over the phone, I have to be cautious whether the service user understands me and I have to repeat and raise my voice and put it in different wording to make sure I got the message across.” – José, Male, Social Worker (Clinician), East London

##### 3.2.2.5 Flexibility in place and space

The benefits of the place in which clinical interactions occurred was described variably by service user participants. On the one hand, some service users described how completing sessions from their own homes would allow them to negotiate what was acceptable within a session and to incorporate self-soothing behaviors such as vaping or having a cup of tea,

*“*Could have a drink or a cup of tea that can be quite settling if you’re upset, I’m suddenly drinking something, gets you a mind off crying or something like that. I think it’s quite comforting be able to have a cup of tea (…) I don’t think you do that in the clinician’s office … And also comforting things around you like I’ve got my cat by my feet.” *-Phoebe, 45-59, Woman, Service User Cornwall*

Whereas others felt that leaving their accommodation and engaging with the community was part of the non-specific therapeutic benefits of community care, and helped create a sense of routine,

“Sometimes changing environments help, innit? Environments contribute to your mood. You know if you’re in your room all day or if you if you have a meeting in the park or something.”- *Clément, 25-34, Man, Service User, East London*

#### 3.2.3 Theme 3: influence of remote care on therapeutic relationships

##### 3.2.3.1 Importance of viewing body language

Service users emphasized the importance of body language and non-verbal cues in conveying meaning during clinical sessions so that the clinician can better understand the emotional state and comfort level of the service user. This was especially important to service users during meetings with their psychiatrists where new diagnoses or changes to their medication could have negative effects.

“They wanted assessment over the phone, and they didn’t see my body shaking and my speech was slurred. So the communication isn’t as transparent I don’t think over the phone. So, I found it quite difficult personally because when someone seeing me face-to-face, they can see what I’m going through” – *Nooriyah, 35-46, Woman, Service User, East London*

Notably, two service users were opposed to sharing their video with their clinician. While they acknowledged the benefits of being able to see the other person’s face and felt that clinicians should keep their cameras on for this purpose, they explained that having to self-monitor their facial expressions and body language made them anxious.

“I understand how it is useful for a clinician. It is just my face, I want to have control over it when I am divulging big chucks of trauma, I don’t want to have to think about my poker face or even think about my face at all really.” –*Radwan, 35-44, Man, Service User, East London*

Clinicians maintained that in-person assessments remained key to best practice to allow for full holistic appraisals. One clinician noted that they would have difficulty picking up on patient engagement or clinical phenomena, such as transference, without access to the rich information provided through body language.

“And again, you can’t pick up like the body language or any sort of transference from the person.” – *Helga, Woman, Clinical Psychologist (Clinician), East London*

While both clinicians and service users felt it was important to be able to see visuals of the other party during their remote sessions, they struggled with the thumbnail video of themselves which many digital communication platforms offer, finding it distracting overall.

##### 3.2.3.2 Acknowledging environmental cues

Service users and clinicians across both sites acknowledged that there was potential for important context cues to be missed when sessions were completed remotely. Service users noted that they could better mask their symptoms and obscure their environment. They explained that the meetings often became dependent on them localizing or describing the presence of these environmental cues which felt unnatural and limited their belief in the utility of the session.

“When I have clinicians come round for meetings [home visit], there is a sense that I can’t lie about what I am doing so when they ask how am I doing and I say well fine and then they will see well 20 diet coke bottles on the floor or the state of my kitchen and they will think well sure, sure you are doing fine?…I think your space is somewhat an extension of you so I think to a certain extent I think it is good for a clinician to view your space.” *– Louis, 25-34, Non-Binary, Service User, East London*

Similarly, clinicians described the cognitive load of having to pay more attention to minute details when online, to improve safeguarding and their consideration of novel issues which are not well defined or where there is a lack of guidance. The presence of children within the home for example was something that had implications for the content of the interaction.

“If you have teenagers and you’re talking about quite sensitive topics, that is a safeguarding issue because you’re exposing those young individuals to quite traumatic discussions … it’s very subtle but they’re constantly having to assess whether that person is safe. So even as I’m sitting, our practitioners are looking to see where am I sitting, is she leaning? Where is her eyeline? But if I lean in really close, you will always find that there are more concerns because you won’t know if there are domestic violence issues, is the perpetrator there? You won’t know.” – *Andrea, Woman, Operational Lead (Clinician), East London*

This prompted important reflections about whether enabling cameras should be mandatory, and what should be visible throughout a session. Clinicians felt that service user cameras should be turned on without any visual manipulations such as blurred backgrounds. Despite this preference, both service users and clinicians felt that the service users’ prerogative should be respected and that this should not be enforced.

Service users, generally, preferred clinicians to have their cameras on as this was seen to help reduce their paranoia as they would be able to confirm their clinician’s identity and look for important cues such as NHS lanyards worn by all staff. Many service user participants expressed being indifferent to the location that clinicians joined them from so long as the space was confidential.

##### 3.2.3.3 Establishing a relationship online

Service users in both sites agreed that they would always prefer to initially meet their clinician in-person. Service users who were open to trying remote care options felt that having the first session in-person would allow them to gauge whether they could imagine remote care being successful and would establish an important level of trust in their clinician. One service user noted that this would be especially important for people who are experiencing paranoia or who were early on in their treatment journey.

“As long as I can see them for the first session and if we have 6 sessions in total I will not need to see their face again in the other sessions because you know you can more or less tell if it is the same person or not through their voice so thankfully I am not at that stage where I would need to see them and hear them and stuff” – *Radwan, 35-44, Man, Service User, East London*

During the clinicians’ focus groups, they explained that building the therapeutic relationship in person was important and established a foundation which could make remote care more effective. Another clinician noted that without this foundation, clinicians completing initial sessions remotely faced the risk of the service user more easily disengaging and the subsequent safeguarding risks with this,

“I’ve only ever worked with people online who I’ve known face-to-face first. So I don’t know how they’ve done it just straight but I think sometimes, for us, that does make a difference. We do have to be quite assertive and turn up. But if it’s online you’ve got that option simply not being online.”*- Kate, Community Psychiatry Nurse (Clinician), Cornwall*

Both clinicians and service user participants therefore felt that sessions should begin face-to-face to allow the therapeutic alliance to be built more naturally.

##### 3.2.3.4 Reducing carer dependence

Service users who were dependent on their carers to attend in-person sessions explained that remote care could help them feel more independent and reduce caregiver burden overall,

“The barrier for me would’ve been my mother taking me by car because I find it hard to go on public transport and she would really, she might have had to cancel an appointment to take me and it would have been difficult sometimes. So if I had it just in my own house it would have been like not having to ask my mother to take me. It would have been easier sometimes”*- Luther, 25-34, Man, Service User, East London*

However, service users who were carers for other family members noted that online care also had the potential to increase carer burden, especially for service users who were older in age and had limited digital literacy. One service user shared that if her elderly mother’s sessions were to be online, she would need to travel to her mother’s home and help set up and support her throughout the entire duration of the call.

While clinicians did not comment on the potential of remote care to reduce carer burden directly, they did remark on the importance of understanding service users’ support networks to mobilize existent strengths. They discussed interactions with carers who might accompany service users during their sessions would be limited.

##### 3.2.3.5 Improving accountability and transparency in care

Service users shared that when they had previously told clinicians that they felt they were approaching a crisis, they were not treated as trustworthy narrators of their own condition because they were well enough to acknowledge that they needed help. Service users considered this type of dismissal as stigmatizing. They felt that if remote care options provided further auditing or documentation of the content of the sessions and what service users had disclosed and discussed, this could help the process of arbitration where there is disagreement between service users and clinicians. Ongoing access to medical records would act as evidence that service users were reliable narrators of their experience.

“There’s just almost stigma where, like if I can acknowledge where I am with my health and I’m not taken serious because it’s like, Oh well, if he can acknowledge that he’s about to have a breakdown or if he can acknowledge that he’s not doing well and he’s aware of this, then there must be nothing wrong with him”*- Clément, 25-34, Man, Service User, East London*

Service users also emphasized the importance of being able to have a clear audit of clinical conversations that occurred, which increased access to clinical notes would allow. Being able to review clinical notes on an ongoing basis would go some way to democratizing the power dynamic between service user and clinician and allow for increased accountability, if there ever was an issue.

“Everyone should be held accountable. I should. They should. Just because you’re in this high powered [position], doesn’t mean you can say that I said stuff that I did not say, that I’ve done this and that and then you should be held accountable if you don’t … So if I’ve got all these proofs [sic, clinical notes] like look well, you never signed on this, it’s proof for myself. And if I if I’m judging myself and questioning my sanity, it makes me look things up and let me check am I going crazy? It would be proof like no you’re not”- *Nooriyah, 35-46, Woman, Service User, East London*

Similarly, clinicians were also committed to increased transparency of clinical notes and care plans for service users. When asked about whether they would be open to features such as automated transcripts being generated, many clinicians across all three focus groups were supportive of such options. One East London clinician noted that this transparency could be quite important to changing the way that service users are written about, noting that the way that current medical notes are written are not always compassionate. However, the potential for increased service user access of clinical notes was seen to be a potential source where there were disagreements between the two parties, which could harm the therapeutic relationship.

“It can really cause difficult conversations, especially if we’re recording somewhere that we wanna keep somebody on medication when they don’t want it, don’t consent. Or if they’re happy and they’ve denied drug or alcohol problems but they’re completely intoxicated or always high or drinking all the time.” *– Dave, Man, Community Mental Health Nurse (Clinician), East London*

#### 3.2.4 Theme 4: desired features

The final theme synthesized data pertaining to improvements, suggestions and features that would be beneficial and desirable to participants. This is not an exhaustive list of proposals but rather a qualitative description of the features that were most oft-cited, particularly regarding care planning software, like DIALOG+ (21).

##### 3.2.4.1 Session reminders

Service users found that they were more likely to miss an appointment due to having forgotten about it if it was remote. This was often because the accompanying appointment reminders were different for remote appointments comparative to in-person ones. They noted that it would be helpful if the processes in place for reminding service users of an in-person appointment were replicated for remote appointments, commenting on the fact that not all service users regularly check their emails.

“If it’s an in-person appointment I’ll get a text reminder obviously saying, oh we’re coming in or whatever. And then, but if it’s the remote things we usually just get emails, so I’d actually have to go on my emails and not miss it.”- *Regina, 18-24, Woman, Service User, Cornwall*

During the interactive workshops, service users who had used NHS apps with medication reminders noted that they had found this function helpful in supporting them to take their medication consistently and on time, and something similar should be available for appointments.

Clinicians also noted that reminders might be helpful in making sure that service users are prepared for their session,

“Maybe a little reminder like you get it on some phone apps, some games, this is best accessed when using headphones. So whether it’s a little reminder to say look, use headphones because they control what they say and if they wanna talk openly about stuff in the middle of Starbucks they can” – *Dave, Man, Community Mental Health Nurse (Clinician), East London*

##### 3.2.4.2 Instructions and pop-up guidance

As service users had varied levels of digital literacy, they emphasized that having an educational or guiding element embedded within digital health tools would be useful. They noted that integration of such a feature would reduce the time commitment to additional training and would make the applications more inclusive overall.

“Like you say you could take people away, chin for days and days and days about how to use software, or you could just have some pop ups on screen like a lot of apps do nowadays, which works well for me.” – *Steven, 45-59, Man, Service User, Cornwall*

Similarly, when asked about what they liked about various digital health apps they had tried in the past, clinicians identified instructional, within-platform pop-up messaging as being very helpful when trying new software.

##### 3.2.4.3 Option for journaling

Service users felt that a journaling feature within remote treatment software would allow service users to be able to reflect and capture their experiences in an accurate, contemporaneous way whilst also providing clinicians with added context. They felt that this might help them share important details without being worried about interruptions related to remote care such as a phone cutting out,

The journaling feature was considered an important tool for recovery which would allow service users to reflect on their progress as well as the day-to-day difficulties they overcome that they might otherwise forget to tell their clinicians about. One service user explained how the journal would not only allow him to acknowledge his resilience but would also permit them to consider what their “vision” for their recovery is,

“I stopped wanting to take showers. I stopped wanting to eat, you know, just normal human things like this and that. And then it’ll be good to like. You know, now I’m acknowledging stuff like that (…) and having to write down things” - *Clément, 25-34, Man, Service User, East London*

Clinicians were supportive of service users having a shareable journal tool but were conscious of the added pressures that this might place on clinicians to read lengthy journal entries between scheduled sessions. Clinicians suggested that service users should be made aware that the journal entries would not be mandatorily read by the clinician between the session but that instead, service users could reference their entries within the session.

##### 3.2.4.4 Transparent summaries of sessions

Service users articulated that having access to session summaries and action items that came from care planning sessions would also allow them to revisit what was discussed in their sessions and would hold clinicians accountable to the commitments that were agreed upon.

One service user explained that often when he saw his clinician, he forgot the advice that was provided previously,

“Let’s see the doctor nine out of 10 times. They can be telling me, like, take this. Take this dude and I’ll just be saying. Yeah, yeah. You know, and you know, that’s why your app will be good to write stuff down and go and back to look at it.” – *Clément, 25-34, Man, Service User, East London*

##### 3.2.4.5 Downloadable & meaningful data

Clinicians in East London felt that it was important for the data to be downloadable in a form that could be analyzed as part of reporting and informatics and be used in a meaningful way to improve service delivery.

“When we talk about delivery of care and I’ll put my hat on as operational lead, we have to understand how this is going to generate data for me to start analyzing.” *– Andrea, Woman, Operational Lead (Clinician), East London*

Similarly, clinicians in Cornwall voiced their frustration with the existing processes in place to monitor service delivery. Namely, they explained that while routine data was collected and this collection interfered with their workflow, the data was never analyzed or findings meaningfully integrated into their practice.

### 3.3 Differences between service user and clinician focus groups

Although there was considerable overlap between the views of clinicians and service users presented above, it is important to identify where there was divergence across the two groups. Ultimately the biggest different was a divergence in where priorities of remote care development should be. Service user priorities largely focused on ensuring the therapeutic relationship was maintained, and that there needed to be consideration around the impact that web-based communication could have on cultural competencies and equity of access. Conversely, clinicians prioritized the efficacy and efficiency of clinical tools, integration into existing clinical systems and the ensuring of safeguarding practices.

## 4 Discussion

Building on previous literature that has identified sub-optimal implementation of remote mental health care as well as negative attitudes towards remote care among service users with psychosis, this focus group study aimed to collect rich qualitative data about the actual experiences of service users and clinicians who have used remotely-delivered care in the past and outline factors influencing the acceptability and adoption of such interventions in community care. Participants were recruited from two NHS Trust sites (Cornwall Partnership NHS Foundation Trust and East London NHS Foundation Trust) in 2021 shortly following the COVID-19 pandemic which provided an opportunistic sample of participants who had some lived experience of using remotely delivered care within NHS community care services. Service users and clinicians highlighted that remote care could not be a complete substitute for face-to-face care, but instead should be provided in combination with in-person care, allowing users to choose what best suited their needs and situation at that time.

Remote sessions provide a useful alternative for users facing specific obstacles to attending in-person such as childcare responsibilities, difficulties in traveling to the clinic, sensory needs, and comorbidities. However remote sessions may not be suitable for patients without access to Wi-Fi, with low digital literacy, those who require an interpreter, in situations where speaking at home may be unsafe or uncomfortable due to shared living conditions, or when the patient experiences paranoia exacerbated by technology use. Recommendations for the design of optimal online care delivery platforms included having a simple, well-constructed interface which is user-friendly, with guiding elements, reminders, session summaries and journaling features were presented. It was also highlighted that beginning a therapeutic relationship with face-to-face sessions first was essential to build the therapeutic alliance and trust.

### 4.1 Comparison with literature

A previous survey study (10) identified that service users with psychosis have high rates of smartphone ownership, only slightly lower than the rates reported in the general population as well as access to appropriate spaces to receive remote care in. This shows that although this patient population may have the means to access remote care, attitudes to accessing remote care are mixed. Previous studies (22–25) on such attitudes have echoed our own findings here that although remote care is a possible treatment, psychosis-related barriers need to be considered. In another survey study (26) it was found that half their sample would prefer use of a mobile application alongside face-to-face support but that one of the biggest barriers to adoption was concerns that such approaches would replace face to face care completely. A quarter also reported that using a smartphone worsened paranoid ideation (26). This aligns with the findings here that remotely delivered community care is not an adequate total substitute for in-person care.

A systematic review in 2019 (24) showed that attitudes and beliefs about digital health interventions were crucial factors, for both staff and service users, in regards to uptake and engagement. Negative attitudes and skepticism led to reduced motivation to engage and see them through to the end (24). Thus, identifying and addressing the presumptions are that are held by service users and clinicians is important. Although logistical aspects (e.g., resources such as device access and Wi-Fi connectivity, staff time etc.) remain integral in ensuring equitable engagement, service transformation needs to also look at how to engage with pervasive attitudes and responses to remote care within this clinical population.

Further, previous studies, particularly those conducted during the COVID-19 pandemic (4, 13, 16), found that remote care often triggers conflicting feelings for people with psychosis and their carers. While some value the convenience offered by such approaches, many acknowledge that remote care adds an element of risk due to the invisibility of non-verbal cues which serve to threaten the development of rapport and a therapeutic relationship, and hampers clinicians’ ability to identify the deterioration of service users’ condition (4). Previous studies have also consistently illustrated the identification of poor internet network infrastructure and training as contributing factors to clinicians’ negative attitudes to remote mental healthcare (4, 13, 16).

Results from a previous survey of over 1,914 service users with severe mental health conditions suggested that for the 8% of participants surveyed who were from minority ethnic communities, there was more openness in engaging with remote mental health services (26). The authors argued that the availability of remote mental health services may increase engagement from minority ethnic communities due to the alleviation of traditional barriers to access such as stigma and transportation costs, and increased therapeutic engagement by including reminders for homework completion (26). However, the service user participants from minority ethnic groups involved in this study communicated their view that remote mental healthcare had the potential to further disenfranchise them within existing healthcare systems. They explained that this was because remote care prevented clinicians from drawing on context cues and body language, leaving more room for cultural bias when assessing clinical presentations. Furthermore, participants were concerned about the availability of interpreters and adequate translations for service users who were not native speakers of English.

The shift to remote healthcare is in accordance with the long-term strategies of the NHS which propose recommendations for the expansion of digital services for greater accessibility and choice (3, 27). However, it is evident from our findings that digital solutions which serve to replicate effective face-to-face care are not adequate alone to encourage service user or clinician engagement. For clinicians, the addition of features to reduce bureaucracy and optimize clinician time are crucial, especially as clinicians have noted that there is a period of adaptation at the beginning of remote service integration whereby they must allocate time and resources to assisting service users who are adapting to the modality (16). Furthermore, consideration for which therapeutic elements remote mental healthcare is most appropriate for is needed. Previous studies have identified that clinicians feel that remote care is not always appropriate for service users in need of human connection or who are at risk for self-harm (4, 28). The most popular types of app suggested by a survey of people with psychosis were symptom monitoring apps, then appointment reminders and then medication reminders (23), with more in-depth, app-based therapeutic interventions less popular. Consistent with this literature, clinicians who participated in our study explained that remote care often compromises their confidence in their risk and safety assessments during clinical contacts. As interest in remote mental healthcare research grows, so will the availability of multidisciplinary research to inform policy. A study sought to address the association of remotely provided therapeutic services with clinical outcomes (29). Integration of such data into NHS long-term plans will be crucial to ensure that best-practice in mental healthcare is iteratively defined and adhered to.

### 4.2 Implications

One of the key objectives of this study was to use the findings to guide the development of a novel software that allows for the remote delivery of DIALOG+, a structured care planning tool used widely in community care within the United Kingdom. Understanding factors influencing acceptability, adaptability, impact on relationships and desirable features for both clinicians and service users is a priority for improving the design, usability, and integration of the software under development. However, the relevance of the findings is not limited to the Remote DIALOG+ software and are transferable to the design of other technologies used by service users with psychotic disorders.

Although there is considerable overlap between the views of clinicians and service users, which is why findings from both groups were presented together here, ultimately there is a divergence in priorities. Service user considerations for remote care uptake largely centered on ensuring the relationship was maintained with the clinician, which included sensitivity to cultural, mental and physical factors. Meanwhile, clinicians were more interested in ensuring the efficacy and efficiency of clinical tools and that safeguarding across modalities was upheld, as well as the maintenance of professional boundaries within sessions. Notably, these priorities are not mutually exclusive within the development of remote interventions for this specific patient population, and it is imperative that NHS plans for remote mental health-care promotion consider the overall message of receptiveness to hybrid models. This is especially important given the NHS’s commitment to upholding patient choice (3). Person-centered approaches which consider and anticipate the role of ethnicity, neurodiversity, and disability in impacting the appropriateness of remote care must also be transparently and comprehensively acknowledged by NHS policies and included within training resources for clinicians.

Overall, further research is needed to understand the therapeutic significance and importance of mechanisms intrinsic to in-person therapeutic interventions such as the behavioral activation implicit to service users’ preparation for and attendance of sessions outside of the home. Moreover, multidisciplinary understandings of how best to adapt clinician working patterns to ensure positive engagement and rapport building online is needed, especially as it pertains to developing cross-cultural understandings of clinical presentations. Remote software should also be compatible with current electronic patient record systems used during face-to-face sessions to ensure consistency across modalities.

Lastly, as service users with psychosis are more likely to experience paranoia, which can be worsened by digital technologies, and can therefore act as a barrier to engagement (26), it is crucial that research advise the development of guidance on how best to introduce and scaffold the use of digital interventions in a way that is conducive to developing positive relationships with services.

### 4.3 Strengths & limitations

A strength of this study is that both clinicians and service users were involved to gain perspectives on remotely delivered mental health care from both groups. Despite them having differing priorities in terms of software development, the general themes were remarkably similar and thus presented together. The research team made use of an opportunistic sample of service users with psychosis who had been recently accessing remotely delivered care, including during the COVID-19 pandemic. We drew on their lived experiences of being offered and receiving remote care to enhance the trustworthiness of the focus group data. As we conducted workshops in both urban (East London) and rural (Cornwall) settings, the study allowed for meaningful comparisons across different service contexts, allowing for improved understanding of the challenges and opportunities in differing geographical settings, particularly in terms of urbanicity. This study successfully engaged service users with varying levels of previous experience of remote care, developing a better understanding of service users’ willingness to engage in remote care with or without previous experience. As part of the workshop-focus groups we also used an exemplar of DIALOG+ as a real-world intervention (which many of the participants would have been familiar with) that could be delivered remotely, to orient the discussion around how that could be developed and improved for frequent use. We believe that this strengthened the validity of the data through grounding the interactive discussion with a concrete example.

However, as data was collected at a time when COVID-19 policies were still in effect, service users and clinicians’ lived experience of remote care as remote delivery was only ever contingent on social distancing policies, therefore tolerance of users may have been greater because it felt like a provisional solution, rather than a long-term replacement. Additionally, the sample included within this study was not representative of the general population, though findings can be transferred to other samples and settings.

## 5 Conclusions

Overall, these findings indicate that both clinicians and service users are willing to incorporate remote care into their treatment as digitalization is becoming an unignorable direction of travel in the transformation and management of the wider NHS. Although there remain concerns from both service users and clinicians, there are potential benefits, uncovered as part of this work, beyond economic efficiency. Clinicians noted that remote care allowed them to schedule clients more flexibly and, in some cases, had enabled clinicians to build rapport with service users who were previously disengaged from in-person care. Service users reflected that the convenience offered by remote care was attractive for certain groups, particularly those who faced barriers to accessing in-person care such as those with childcaring or working responsibilities. However, all participants strongly felt that in-person community care cannot be wholly replaced by digital platforms and remote care delivery. Both service users’ and clinicians’ requirements for remote care, which have been outlined within this article, need to be addressed through suitable software development and eventual adoption. As a basis, reliable internet access, improved training, and more regulated reporting of clinical outcomes for remote care need to be developed and standardized across different modalities to allow for more hybrid approaches to be used. More widely, further research is needed to consider how both specific and non-specific factors of mental health care may be replicated within digital communication platforms and remote care may impact on treatment outcomes.

## Data Availability

The datasets presented in this article are not readily available because of the possibility of participant identification due to the nature of the data (i.e., in-depth qualitative data). Request to access the datasets should be directed to the corresponding author.
