# Supplementary material for: Exploring the acceptability of remote care for people with psychotic disorders in the community: practical challenges and desired features
Source: Front Psychiatry. 2025 Nov 3;16:1409455. doi: 10.3389/fpsyt.2025.1409455 (PMC12620910; doi:10.3389/fpsyt.2025.1409455)
Supplement: Supplementary file 1 [file SupplementaryFile1.docx]

Figure 1

*Service User Topic Guide for Focus Groups*

**In the workshop you saw DIALOG+ and examples of remote health care delivery, what do you think of these apps?**

- What do you like/dislike?
- Have you used any of these apps that were shown in the workshop before?
- If you haven’t used any of these apps shown, from what you saw in the demonstration do you think they could be helpful to you or not?
- If you were offered Remote DIALOG+ by your treatment team what would your initial thoughts be?

**[GENERAL VIEWS OF REMOTE CARE/ EXPERIENCES]**

**Do you think it is useful for your care to be delivered remotely?**

- What do you think is beneficial about remote care?
- What do you think are the things that will stop you from using barriers to usage?
- What could be done to improve remote care, or make it more attractive?
- What changes do you think remote care would make to your current treatment?
- What are the key aspects of your care that would need to be available (replicated) when accessing remote care?

**What would make remote care meetings easier or more difficult with your clinician?**

**[PAST EXPERIENCES OF REMOTE CARE]**

**Many people would have received remote care during COVID-19, what was your experience of that?**

- What were some of the biggest issues in receiving treatment in this way?
- What did you think the biggest obstacles for the clinicians were in delivering remote care during the pandemic?
- From your past experiences using remote healthcare, do you feel it is important to see your clinicians face on the screen all the time in your meetings?
- Did you find remote delivery of care during this time useful or helpful?

**[INTERFACE]**

- When you had remote healthcare in the past, were you able to see your face and your clinicians on the same screen?
  - Did you like seeing both your faces on the screen at the same time?
- In the past when you had your care meetings online, what sort of device did you use, was it your smartphone, a tablet, a laptop etc?
  - Do you think a phone screen is big enough to use for these meetings?
- When you had these care meetings online, did you feel your care coordinator was repeating themselves a lot?
  - Do you think it would be helpful in the future to be able to skip certain areas of the meetings to avoid the care coordinator repeating themselves?
- In the past, what area of your care meetings took the longest?

**[CONTEXT OF CONSULTATION]**

**Can you describe the room or space that you would want to engage in remote care?**

- Would you feel comfortable doing this from home?
- What are some of the issues that you think would impact on the interaction (noise, privacy etc)?
- Would you want anyone else in the room with you?
- Can you describe the environment that you would want to see your clinician in?

**[INFORMATION GOVERNANCE/ PRIVACY]**

**What concerns do you have with your information/data being discussed over an app?**

- How could we overcome these concerns, if possible?
- Are you willing to openly discuss private and/or sensitive things with your clinician when not face to face?
- What could the clinician do to make you feel more protected?

**[ACCESS]**

**Remote meetings can be conducted using different electronic devices, such as smartphones etc…which of these resources do you have access to? (such as smartphone, tablet or laptop)**

- What resources do you not have access to?
- What are the barriers to having access to them?
- How could we overcome these barriers?
- What type of support would be helpful?

**[COMPETENCY]**

**Do you have an example of a digital app that you don’t feel confident in using?**

- Why do you think that is?
- How could we improve your confidence in using that app?
- Are there any apps that you do feel confident in using?
- What is it about those apps that make you feel confident in using them?

**[TRAINING]**

**Do you believe training is necessary for using software that helps with remote delivery of care?**

- Who would you want to deliver your training?
- Would you want that training in person or online?
- Would it be useful to be provided with a training manual/ leaflet on how to use these digital apps?

**[FORMING RELATIONSHIPS]**

**How do you feel about establishing a new relationship with a clinician online?**

- Do you think any key things will be lost when working online with a clinician (e.g. body language, personality traits etc)?
- Do you believe the relationships you have with clinicians you have only met online are as strong as the relationship with clinicians you have met in person?

**If you had one key concern about remote care, what would that be?**

**Having had this discussion, If you were offered Remote DIALOG+ now by your treatment team/ clinician what questions would you have?**
