# Supplementary material for: Exploring the acceptability of remote care for people with psychotic disorders in the community: practical challenges and desired features
Source: Front Psychiatry. 2025 Nov 3;16:1409455. doi: 10.3389/fpsyt.2025.1409455 (PMC12620910; doi:10.3389/fpsyt.2025.1409455)
Supplement: Supplementary file 2 [file SupplementaryFile2.docx]

Figure 2

*Clinician Topic Guide for Focus Groups*

**In the workshop you saw DIALOG+ and examples of remote health care delivery currently in use in the NHS, what do you think of these apps?**

- What did you like/dislike?
- Have you used any of these apps that were shown in the workshop before?
- In your opinion of using one of the apps shown, were these apps helpful or not?
  - If you haven’t used any of these apps shown, from what you saw in the demonstration do you think they could be helpful to you or not?
- If you were encouraged to deliver Remote DIALOG+ to your patients what would your initial thoughts be?

**[GENERAL VIEWS OF REMOTE CARE/ EXPERIENCES]**

**Do you think it is useful for mental health services to use remote care?**

- What do you think is beneficial about remote care from a clinician perspective?
- What do you think is beneficial about remote care from a patient perspective?
- What do you think some of the implementation barriers are?

**[PAST EXPERIENCES OF REMOTE CARE]**

**From your past experiences of delivering care remotely to patients, how did you find delivering care online?**

**Drawing on your experience of delivering remote care during COVID-19 what could be done to improve delivery of remote care?**

- Do you feel it is important to see your patients’ face and/or surroundings on the screen at all times in your meetings?
- In the past, were you able to see your face and your patients face on the same screen at the same time?
  - Do you believe that the shared screen format of both your faces worked well?
- In the past when you delivered your care meetings online, what sort of device did you use (smartphone, a tablet, a laptop etc?
  - Do you think a phone screen is big enough to use for these meetings?
- When you delivered health care remotely, did you feel that there was a lot of repetition within these meetings?
  - Do you think it would be beneficial to be able to skip certain areas of the meetings to avoid repetition?
- From your previous experience of using remote health care, did you feel the delivery of care was compromised by implementing it online or not?
- How did you utilise your time implementing the care remotely?
  - In your opinion, did you feel you were more time-efficient delivering care online to in-person?
- In your opinion, what were the challenges you faced delivering care online? Were these challenges more practical based (such as poor wifi, limited resources, lack of teaching to implement care online) or were they challenges in terms of patient engagement and forming a therapeutic alliance?

**[TRAINING]**

**How much training do you think you will require to use new software for the remote delivery of care?**

- How confident do you feel in delivering remote care currently?
- What areas of routine care do you believe needs to be a focus of the training? (e.g. how to conduct risk assessments online; how to prescribe medications online without being able to assess the patient in person; how to navigate the software?)
- Do you believe it would be beneficial to have workshops and role plays in how you would deliver remote healthcare to patients?
- Who would you want to deliver your training?
- Would you want that training in person or online?
- Do you believe it is necessary for patients to receive training to use such technology?

**[CONTEXT OF CONSULTATION]**

**Can you describe the room or space that you would want to engage in remote care?**

- Would you feel comfortable doing this from your home?
- What are some of the issues that you think would impact on the interaction (noise, privacy etc)?

**Can you describe the environment that you would want to see your patient in?**

- How does this impact on safeguarding measures?

**[INFORMATION GOVERNANCE/ PRIVACY]**

**Do you have any concerns in regards to your patients' information/data being discussed over an app?**

- What concerns do you have?
- How could we overcome these concerns, if possible?
- From past experiences, do you believe your patients are willing to openly discuss private and/or sensitive things with you when not in person?
- What could be done, do you believe, that would make your patients feel more protected when using an app?

**[SAFEGUARDING]**

**Do you have any concerns about safeguarding when it comes to remote delivery of care?**

- How would you create adequate safety plans when working remotely?
- What issues have come up in the past?

**[ACCESS]**

**Do you have access to the resources (such as smartphone, tablet or laptop) needed to carry out a remote care meeting?**

- What resources do you not have access to?
- What type of ongoing support or supervision would be helpful?

**[EXPERIENCE OF TECHNOLOGY/ APPS]**

**In the past, if you have used digital apps, did you feel confident in using them?**

- What areas did you not feel confident in?
- How could we improve this?
- Is there anything that these apps missed that you believe would be beneficial to be implemented in future apps?

**[COMPETENCY]**

**Do you have an example of a mobile app that you don’t feel confident in using and/or implementing either personally or with your patients?**

- Why do you think that is?
- How could we improve your confidence in using that app?

**[FORMING RELATIONSHIPS/THERAPEUTIC ALLIANCE]**

**How do you feel about establishing a new relationship with a patient online?**

- Do you think any key things will be lost when working online with a patient (e.g. body language, personality traits etc)?
- Do you believe the relationships you have with patients you have only met online are as strong as the relationship with patients you have met in person?
- From past experiences of delivering digital consultations, do you believe it is more difficult to assess patients capacity issues remotely compared to in-person?
- From your previous experience of delivering remote healthcare, do you believe it is easier to create and maintain a therapeutic alliance if you use a digital platform that allows you to visually see your patient?

**If you had one key concern about remote care, what would that be?**

**Having had this discussion, If you were offered Remote DIALOG+ now to deliver to your patients what questions would you have?**
