# Supplementary material for: Exploring the acceptability of remote care for people with psychotic disorders in the community: practical challenges and desired features
Source: Front Psychiatry. 2025 Nov 3;16:1409455. doi: 10.3389/fpsyt.2025.1409455 (PMC12620910; doi:10.3389/fpsyt.2025.1409455)
Supplement: Supplementary file 3 [file Table1.docx]

Table 1:

*List of (self-reported) professional backgrounds of clinician participants*

|  | | | |
| --- | --- | --- | --- |
| **Focus Group** | **No.** | **Location** | **Professional Background** |
| *East London FG 1* | 1 | East London | Clinical Psychologist |
|  | 2 | East London | Community Psychiatry Nurse |
|  | 3 | East London | Mental Health Social Worker |
|  | 4 | East London | Mental Health Nurse |
|  | 5 | East London | Support Worker |
|  | 6 | East London | Social Worker |
|  | 7 | East London | Community Mental Health Nurse |
| *East London FG 2* | 8 | East London | Operational Lead |
|  | 9 | East London | Mental Health Social Worker |
|  | 10 | East London | Social Worker |
|  | 11 | East London | Community Psychiatry Nurse |
|  | 12 | East London | Support Worker |
|  | 13 | East London | Support Worker |
|  | 14 | East London | Care Coordinator |
| *Cornwall FG 1* | 15 | Cornwall | Community Psychiatry Nurse |
|  | 16 | Cornwall | Care Coordinator (Early Intervention in Psychosis service) |
|  | 17 | Cornwall | Care Coordinator |
|  | 18 | Cornwall | Nurse |
|  | 19 | Cornwall | Support Worker |
|  | 20 | Cornwall | Care Coordinator |
|  | 21 | Cornwall | Team Manager |
|  | | | |
