# Supplementary material for: Exploring the acceptability of remote care for people with psychotic disorders in the community: practical challenges and desired features
Source: Front Psychiatry. 2025 Nov 3;16:1409455. doi: 10.3389/fpsyt.2025.1409455 (PMC12620910; doi:10.3389/fpsyt.2025.1409455)
Supplement: Supplementary file 4 [file Table2.docx]

Table 2

*Overview and descriptions of themes and subthemes*

|  | | |
| --- | --- | --- |
| Theme Name | Subtheme Name | Subtheme Description |
| Theme 1: Factors Influencing the Acceptability of Remote Care | 1.1 Willingness to Engage in Remote Care | Remote care is not an adequate complete substitute for face-to-face care and as such, face-to-face care is always necessary in some capacity for treatment delivery to be effective. However, remote care might be especially helpful for those who have significant childcare responsibilities, social anxiety or unique sensory needs, or where there are high-cost implications or time requirements to travel to clinics. |
|  | 1.2 Access to Devices, and Connectivity | Many participants expressed that poor internet connectivity in the home and lacking access to functional digital devices would hamper their ability to engage in remote care even if they wanted to. |
|  | 1.3 Digital Literacy and Access to Support | For remote care to be effective, service users must be able to understand how to use it. Supporting service users so they feel able to identify and resolve commonly experienced issues is of paramount importance. |
|  | 1.4 Privacy, Security and Safety | During remote care sessions, assurances of privacy are difficult to have due to the possibility of others (family, neighbours, etc.) being able to hear interactions. This discourages service users from being free to fully discuss what they want to. A private space is therefore needed. |
|  | 1.5 Previous Experiences | Those who had poor prior experiences of remote care such as misdiagnosis or a feeling of disconnection from their clinicians are less likely to consider it as an option in the future. Comparably, those who have had positive experiences view it as being more viable. |
| Theme 2: Adaptability of Remote Care for Inclusivity | 2.1 Appropriateness of Remote Care Across Illness Severity | Digital paranoia is often experienced by service users with psychosis. If introduced when delusions or auditory hallucinations are active, remote care can become a source of distress in itself. Remote care should be adapted and gradually introduced based on each person’s comfort level and symptom severity. |
|  | 2.2 Adjustments to Remote Care for People with Multiple Conditions | Remote care needs to consider the accessibility needs of those with disabilities. Remote care needs to be equitable and must anticipate differences such as visual impairment, hearing impairment or cognitive difficulties. |
|  | 2.3 Importance of Simplified Language and User-Friendly Design | Simple language that is easy to follow allows those with psychosis the ability to focus and engage within their sessions. |
|  | 2.4 Cultural Inclusivity and the Importance of Including Non-English Speakers | People from different cultural backgrounds will engage in remote care differently and clinicians must be cautious when drawing conclusions based on elements such as volume of speech. Remote care can remove cues such as body language which can make communication with those who have limited English easier in face-to-face contexts. Remote care options should be translated to meet the needs of service users wanting to be engaged in their care. |
|  | 2.5 Flexibility in Place and Space | Leaving the house to visit a new space can be therapeutic for some service users. While remote care may enable service users to remove this element from their routine, service users can choose the locale or environment they join from. Remote care also allows service users to engage in other soothing behaviours such as stroking one’s pet, holding a warm cup of tea, or vaping, giving them more tools to control their anxieties surrounding accessing care. |
| Theme 3:  Influence of Remote Care on Therapeutic Relationships | 3.1 Importance of Viewing Body Language | It is important for clinicians to be able to see body language to get a more holistic idea of the client’s presentation. Video (or visual) conferencing is preferable to phone conversations for this reason. |
|  | 3.2 Acknowledging Environmental Cues | Remote care can conceal factors such as personal hygiene or the environment the service user is in. This can make it more difficult for the clinician to evaluate risks. Service users felt confident that they would be better able to mask their symptoms if they were using remote care. Such concealment can have a negative impact on service users in need of care or safeguarding and can impact their relationship with treatment and help-seeking overall. |
|  | 3.3 Establishing a Relationship Online | Building a relationship with a clinician can be a gradual and lengthy process. Service users felt that meeting the clinician in-person first and having a pre-established relationship was crucial before remote care could begin. |
|  | 3.4 Reducing Carer Dependence | Remote care can help those who are dependent on carers for transportation to appointments to become more independent and self-reliant, improving their sense of empowerment overall. |
|  | 3.5 Improving Accountability and Transparency in Care | Service users viewed remote care as a tool for democratising power within the therapeutic relationship. By having a digital paper trail of service user and clinician engagement, service users could feel empowered to challenge clinician misconduct when it occurred. Clinicians were supportive of increased transparency but noted the potential for ruptures in the therapeutic relationship if all care notes were able to be viewed by service users. |
| Theme 4: Desired Features | 4.1 Session Reminders | In-person appointments are generally accompanied by several reminders by phone from the CMHT to make each service user aware of their upcoming appointment. However, remote appointments are often accompanied by an e-mail reminder which assumes that the service user will check this account regularly. Session reminders via phone are needed for remote appointments, as well as face-to-face ones. |
|  | 4.2 Instructions and Pop-Up Guidance | Inter-platform instructions or “pop-ups" to remind users of the features and sequential steps of the remote care procedure can help engage service users without being deterred by time consuming training manuals and events. |
|  | 4.3 Option for Journaling | Having a digital journal feature which can be completed between sessions would remind service users of the key points that they wanted to raise during their session but often forget about. This can also be an outlet for therapeutic thinking and reflection, assisting service user to process their experiences of care. |
|  | 4.4 Transparent Summaries of Sessions | Being able to view session notes and action points will allow service users to be more engaged during the session, as they will not need to direct their energy toward taking their own notes during the session. This also encourages clinician accountability and can be a helpful tool to reference when experiencing delusions. |
|  | 4.5 Downloadable and Meaningful Data | Clinicians wanted to be able to download data so that the sessions could be used to advise quality improvement efforts on a team and Trust level. |
|  | | |
